# X chromosome inactivation skewing is common in advanced carotid atherosclerotic lesions in females and predicts secondary peripheral artery events

**DOI:** 10.1186/s13293-023-00527-6

**Published:** 2023-07-05

**Authors:** Michele F. Buono, Ernest Diez Benavente, Mark Daniels, Barend M. Mol, Joost M. Mekke, Gert J. de Borst, Dominique P. V. de Kleijn, Sander W. van der Laan, Gerard Pasterkamp, Charlotte Onland-Moret, Michal Mokry, Hester M. den Ruijter

**Affiliations:** 1 Laboratory of Experimental Cardiology, University Medical Center Utrecht, Utrecht University, Utrecht, The Netherlands; 2 Department of Vascular Surgery, University Medical Center Utrecht, Utrecht University, Utrecht, The Netherlands; 3 Central Diagnostics Laboratory, University Medical Center Utrecht, Utrecht University, Utrecht, The Netherlands

**Keywords:** Atherosclerosis, Sex-differences, XCI skewing, Vascular, Carotid, Peripheral

## Abstract

**Background and aim:**

Sex differences in atherosclerosis have been described with female plaques being mostly perceived as stable and fibrous. Sex-specific mechanisms such as mosaic loss of the Y chromosome in men have been linked to cardiovascular health. In women, X-linked mechanisms such as X chromosome inactivation (XCI) skewing is common in several tissues. Yet, information on the role of XCI in female atherosclerotic plaques is lacking. Here, we investigated the presence of XCI skewing in advanced atherosclerotic lesions and its association with cardiovascular risk factors, histological plaque data, and clinical data.

**Methods:**

XCI skewing was quantified in 154 atherosclerotic plaque and 55 blood DNA samples of women included in the Athero-Express study. The skewing status was determined performing the HUMARA assay. Then, we studied the relationship of XCI skewing in female plaque and cardiovascular risk factors using regression models. In addition, we studied if plaque XCI predicted plaque composition, and adverse events during 3-years follow-up using Cox proportional hazard models.

**Results:**

XCI skewing was detected in 76 of 154 (49.4%) plaques and in 27 of 55 (67%) blood samples. None of the clinical risk factors were associated with plaque skewing. Plaque skewing was more often detected in plaques with a plaque hemorrhage (OR [95% CI]: 1.44 [1.06–1.98], P = 0.02). Moreover, skewed plaques were not associated with a higher incidence of composite and major events but were specifically associated with peripheral artery events during a 3-year follow-up period in a multivariate model (HR [95%CI]: 1.46 [1.09–1.97]; P = 0.007).

**Conclusions:**

XCI skewing is common in carotid plaques of females and is predictive for the occurrence of peripheral artery events within 3 years after carotid endarterectomy.

**Supplementary Information:**

The online version contains supplementary material available at 10.1186/s13293-023-00527-6.

## Plain language summary

Sex-differences have been observed in the development of atherosclerosis between men and women. Women tend to have more stable and fibrous plaques compared to men. Sex-specific mechanisms such as mosaic loss of the Y chromosome in men, were associated with cardiovascular health. In women, despite X-linked mechanisms like X chromosome inactivation (XCI) skewing was identified in various tissues. However, its relationship with atherosclerosis has not yet been investigated. In our study, we explored if prevalence of XCI skewing in advanced atherosclerotic lesions related to cardiovascular risk factors, histological plaque data, and clinical information. We found that XCI skewing was present in approximately 50% of human plaques, particularly those with plaque hemorrhage. Interestingly, we did not find any notable relationship between plaque skewing and clinical risk factors. However, we found that XCI was more present in women with peripheral artery events during the 3 years period following carotid endarterectomy. In summary, our findings indicate that XCI skewing is commonly observed in carotid plaques among females and may serve as a predictive factor for the occurrence of peripheral artery events within 3 years after carotid endarterectomy.

**Supplementary Information:**

The online version contains supplementary material available at 10.1186/s13293-023-00527-6.

## Highlights

- XCI skewing is common in half of the carotid plaques of females and mostly in those plaques with a plaque hemorrhage.
- None of the clinical risk factors were associated with plaque skewing.
- Skewed plaques were specifically associated with peripheral artery events during a 3-year follow-up period after carotid endarterectomy.

**Supplementary Information:**

The online version contains supplementary material available at 10.1186/s13293-023-00527-6.

## Introduction

Atherosclerosis is often the underlying pathology for cardiovascular disease, characterized as a chronic and progressive inflammatory condition in which oxidized low-density lipoproteins infiltrate into the vascular wall, followed by inflammatory cells and leading to the formation of atherosclerotic plaques [1]. These plaques present significant phenotypic differences between the sexes [2]. Females have a more fibrous phenotype, more prone to erode while in men a vulnerable rupture-prone phenotype is more often reported [3, 4]. The differences in the clinical presentation of atherosclerosis between males and females are mainly attributed to sex hormones and sex chromosomes [5].

Females have two X chromosomes, one active (X_a_) and one inactive (X_i_) to ensure that the expression levels of X-linked genes are similar between females and males [6]. This dosage compensation mechanism is known as X-chromosome inactivation (XCI). Briefly, XCI is a process regulated by the expression of XIST on the X_i_ [7], and it is thought to start as early as the eight-cell stage during human embryonic development [8]. The inactivation of one of the parental X chromosomes seems to randomly occur within each cell during preimplantation phases and it gets mitotically inherited by all the future somatic daughter cells [6, 9]. This random inactivation results in a mosaic of cells within female tissues, where both parental X-linked alleles are expected to have balanced expression (50:50). The inactivation of a particular parental X is translated with an unbalanced expression and known as XCI skewing or nonrandom XCI [9].

The preferential X inactivation (XCI skewing) is the result of multiple mechanisms that can either take place directly during embryonic development (primary skewing) [10] or later in life (acquired or secondary skewing) due to positive selection of cells that after having inactivated a particular parental X, acquire a survival advantage over cells who inactivated the other parental X chromosome [9]. Twin studies have reported that genetic factors contribute to XCI skewing (primary) in blood-derived cells [11, 12], while other studies indicated that most of the XCI skewing levels in human are acquired secondarily [13], associated with ageing [11, 14–18] and frequent in the healthy and diseased female tissues [19]. Besides, females affected by X-linked disease [20–22], autoimmune disorders [23, 24] and cancers [25, 26] commonly present XCI skewing.

Hence, given the lack of information on X-linked mechanisms in female atherosclerosis, we investigated the prevalence of XCI skewing in advanced atherosclerotic plaque and blood of female patients who underwent carotid endarterectomy (CEA). Then, we studied if age and cardiovascular risk factors were related to plaque skewing. We also studied if plaque skewing was related to histological plaque characteristics and with an increased risk of having secondary cardiovascular events during 3-years follow-up.

## Materials and methods

### Patient characteristics

The Athero-Express biobank study is an ongoing cohort study that includes atherosclerotic plaques and blood of patients undergoing either carotid or femoral endarterectomy in 2 large tertiary referral hospitals (University Medical Center Utrecht and St. Antonius Hospital Nieuwegein) in the Netherlands. The patients used for this study have been selected based on the availability of the DNA samples isolated from carotid plaque and matched blood of female donors. Clinical data were obtained from medical files and standardized questionnaires. Age was determined as age at surgery. Current smoking was determined as patient-reported smoking in the past year. Hypertension and hypercholesterolemia were self-reported. Diabetes mellitus was considered present in any of the following cases: use of insulin or oral glucose inhibitors, self-reported diabetes mellitus in the patient questionnaire, or diabetes mellitus extracted from the medical file. A history of coronary artery disease was considered present if the patient had experienced a myocardial infarction or underwent a percutaneous coronary intervention or coronary artery bypass grafting surgery. Peripheral arterial occlusive disease was considered present if the patient either presented with an ankle-brachial index < 0.7, claudication complaints, or underwent percutaneous or surgical intervention for peripheral arterial occlusive disease.

Follow-up was obtained by questionnaires sent to the patients by mail 1, 2, and 3 years post-operatively. Composite events include myocardial infarction (MI), hemorrhagic and ischemic stroke, coronary angioplasty, peripheral intervention, cardiovascular death, coronary bypass, leg amputation, sudden death, fatal aneurysm rupture, other cardiovascular death. Major adverse cardiovascular events (MACE) include MI, hemorrhagic and ischemic stroke, cardiovascular death, sudden death, fatal heart failure fatal aneurysm rupture, other cardiovascular death. Peripheral artery events include percutaneous transluminal angioplasty (PTA), peripheral (re-) intervention of the limb arteries, and leg amputation [27]. All the endpoints were validated using medical records. The medical ethics boards of both hospitals approved of the study, which is conducted in accordance with the declaration of Helsinki, and the subjects gave informed consent.

### Sample collection

Blood was obtained before surgery and plasma was subsequently stored at − 80 °C. Plaque specimens were immediately processed after removal during surgery. After identification of the area with the largest plaque burden (culprit lesion), the plaque was cut transversely into segments of 5 mm. The culprit lesion was fixed in 4% formaldehyde and subsequently decalcified and embedded in paraffin. Cross-sections were stained for histological examination. Remaining segments were stored at − 80 °C and used for the measurement of other paraments (e.g. inflammatory cytokines) and isolation of DNA. A detailed description of the samples phenotyping within the Athero-Express study can be found elsewhere [28].

### HUMARA assay

The HUMARA assay was performed on the DNA isolated from blood and plaque as following. Working DNA solutions were prepared by diluting 10 × with DNAse free H2O and added, in an equal volume, to a 2 × enzyme reaction mix (2 × NH4 Taq buffer, 3u *HpaII* (NEB) and 4 mM MgCl2) and incubated at 37 °C of 1 h. then, an equal volume of PCR reaction mix (1 × NH4 Taq buffer, 1.5 mM MgCl2, 0.8 mM dNTP, 1uM Humara primer mix (fw = 5′-ACCGAGGAGCTTTCC AGAAT-3′; rv = 5′-TGGGGAGAACCATCCTCAC-3′) [29] and 1uM GAPDH primer mix (fw = 5′-CGCAGGCCGGATGTGTTC-3′; rv = 5′-ACACACACGCCTCCCCTC-3′), 0.25u Hotstart Taq polymerase (NEB) and 5% DMSO), was added to the restriction mix and a standard PCR program with a 60 °C annealing temperature and 13 cycles was performed. The PCR mix was diluted 2.5 × with MQ.

A second PCR reaction performed by adding 5ul of the diluted PCR mix to 15ul of reaction mix (1 × NH4 Taq buffer, 3 mM MgCl2, 0.8 mM dNTP, 1 µM HUMARA primer mix and 1 µM GAPDH primer mix, 0.25u Hotstart Taq polymerase and 5% DMSO).

A standard PCR program with a 60 °C annealing temperature and 15 cycles was performed. Samples were analyzed on 8% PAGE gel running at 200 V for 100 min and visualized with sybr safe.

### Quantification of XCI skewing

The quantification of the XCI skewing levels has been assessed by measuring the band intensities after HUMARA assay. Interpretation of PCR results from each case required the following criteria: (1) successful PCR pre-amplification and amplification of DNA, both undigested and *HpaII*-digested; and (2) presence of two different androgen receptor alleles (heterozygous). An internal homozygous control, GAPDH containing *HpaII* digestion sites to exclude experimental artifact of incomplete enzyme digestion was included in each reaction. If GAPDH control showed undigested DNA, the reaction was considered as failed, repeated and if the similar result was obtained, the sample was removed from the analysis. Each reaction gave two intensity percentages (one per allele), the one equal or greater than 50% was selected and used in our data to determine presence of skewed XCI.

### Determination of plaque skewing

Dichotomous plaque skewing variable was determined by distribution method using bestNormalize package in R Studio V. 1.1.456. The cut-off used to determine skewed plaque was if greater than 63.9% (Additional file 1: Fig. S1).

Binned plaque skewing variable has been defined by four skewing levels. The plaques having skewing percentage < 60% were defined as non-skewed (No, 37%), ≥ 60% and < 70%, as lowly skewed (Low, 30%), ≥ 70% and < 80% as skewed (Mid, 18%), and ≥ 80% as highly skewed (High, 15%).

### Statistical analyses

XCI plaque skewing variables were associated with baseline characteristics. To determine possible confounders χ^2^ (categorical variables), ANOVA (continuous variables), and Kruskal–Wallis (non-normal continuous variables) tests, where applicable, were used.

Histological plaque characteristic variables (fat content, amount of calcification, collagen, presence of plaque hemorrhage, macrophage, smooth muscle cell, neo-vessel and glycophorin content) were used to assess whether plaque skewing associates with any of those plaque characteristics. We performed Saphiro test on the continuous variables to determine their distribution. All variables having Saphiro test with P ≤ 0.05 were considered non-normal distributed. These non-normal distributed variables (e.g. Glycophorin content) were transformed using BestNormalize package in R Studio to achieve normal distribution and used as outcomes in linear regression models. The transformation was based on the order quantile normalization technique and it was achieved applying the following transformation: g(x) = Φ − 1(rank(x) − 1/2 / length(x)) where x refer to the original data. The continuous variables that cannot be normalized (e.g. neo-vessel content) were binarized using BestNormalize package in R Studio and used as dichotomous outcomes for logistic regression.

The plaque characteristics (e.g. plaque hemorrhage) associating with plaque skewing were used to assess whether classical cardiovascular risk factors influence the association of plaque skewing with plaque characteristics. The significance of the association in the logistic regression models was assessed using Wald test and a P ≤ 0.05 was considered significant.

Kaplan–Meier, for univariate curves, and cox proportional hazard regression, for adjusted curves for all covariates that associated with outcome, methods were used to investigate the association between plaque skewing and secondary cardiovascular events (composite, major adverse cardiovascular and peripheral artery events) during 3-year follow-up. Statistical significance was assessed using Wald test and a P ≤ 0.05. Global statistical significance of the model was assessed using Mantel–Cox (log-rank) test. All statistical analyses were performed using R statistical package v. 4.1.2 within R Studio v. 1.1.456.

### Differential expression analysis

Read counts obtained from RNA sequencing data and DESeq2 package in R were used for differential gene expression analysis between groups. Genes meeting the following criteria were considered as differentially expressed genes (DEGs): a P-value < 10^–6^ and a Log2 fold change ≥ 0.5 (up-) and ≤ − 0.5 (down-regulated). The volcano plot was generated using the “EnhancedVolcano” package in R. The genes that exhibited significant up- and down-regulation relative to the first condition (e.g., First Condition vs Second Condition) are depicted as red dots on the right and left sides, respectively.

## Results

### Plaque XCI skewing is highly prevalent in female atherosclerotic patients

We selected all female atherosclerotic patients who underwent carotid endarterectomy (CEA) and had DNA stored for XCI determination (n = 154). Of these 154 women, a subset of 55 women had DNA stored from blood samples. Baseline characteristics are displayed in Table 1. In short, these women were 66 years old on average and had a median BMI of 26 at baseline. At the day of the hospitalization, 32% of the patients had a previous history of peripheral artery occlusive disease (PAOD), while 26% had a previous history of coronary artery disease (CAD). Most of the patients suffered from hypertension (88%) and/or hypercholesterolemia (70%) and were on antiplatelet (94%) and/or lipid-lowering (77%) medications. Also, 71 patients (47%) reported to smoke and 19% had diabetes.

**Table 1:** Baseline characteristics of patients with and without XCI skewing in plaque

XCI skewing	Overall	No Skewed	Skewed	P value
N (%)	154 (100%)	78 (50.6)	76 (49.4)	–
Age in years (mean, SD)	66 (9)	66 (9)	67 (9)	0.40
BMI (median, IQR)	26 [23, 28]	26 [23, 28]	25 [23, 27]	0.26
Current smoker, yes (%)	71 (47)	40 (53)	31 (41)	0.22
Diabetes mellitus, yes (%)	29 (19)	15 (19)	14 (18)	1.00
Hypertension, yes (%)	135 (88)	71 (91)	64 (84)	0.30
Hypercholesterolemia, yes (%)	105 (70)	51 (67)	54 (72)	0.63
History of coronary artery disease (%)	40 (26)	18 (23)	22 (29)	0.52
History of PAOD (%)	50 (32)	22 (28)	28 (37)	0.33
Use of antiplatelet therapy (%)	144 (94)	71 (91)	73 (96)	0.35
Use of lipid-lowering drugs (%)	119 (77)	58 (74)	61 (80)	0.50
GFR (MDRD) mL/min per 1.73 m2 (mean, SD)	70 (20)	69 (20)	71 (19)	0.41
LDL in mg/dL (median, IQR)	111 [82, 145]	109 [80, 144]	111 [82, 144]	0.90
HDL in mg/dL (median, IQR)	46 [37, 55]	45 [37, 55]	46 [37, 60]	0.80
Total cholesterol in mg/dL (median, IQR)	189 [147, 228]	185 [147, 226]	203 [147, 230]	0.78
Triglyceride levels in mg/dL (median, IQR)	127 [94, 189]	127 [96, 187]	129 [89, 197]	0.76
**Presenting symptoms (%)**				0.25
Asymptomatic	25 (18)	15 (21)	10 (16)	
Transient Ischemic Attack (TIA)	77 (57)	36 (50)	41 (64)	
Stroke	34 (25)	21 (29)	13 (20)	

**Fig. 1:**
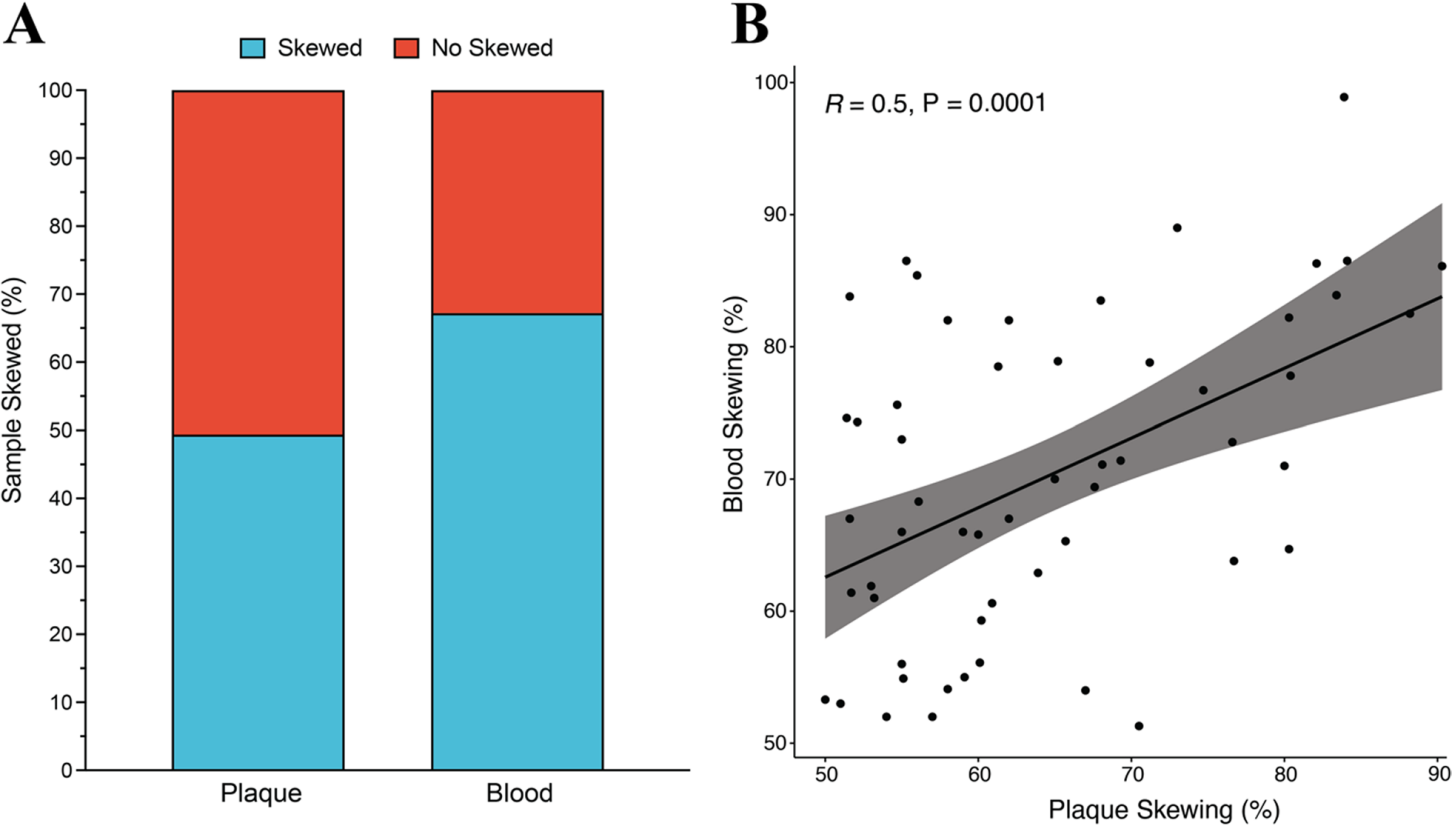
**A** Bar plot showing the occurrence of skewing in percentage in plaque (49.4%) and blood (67.2%). **B** Scatter plot showing strong association between plaque and blood skewing (P = 0.0001)

XCI skewing was determined in 154 carotid plaque and 55 matched blood DNA samples by performing a HUMARA assay followed by PAGE gel quantification. We used these quantitative data to determine XCI skewing status using a computed cut-off value of 63.9% (Additional file 1: Fig. S1). XCI skewing occurred in 76 of 154 plaques (49.4%), while in blood, it was higher with 67.2% (37/55) (Fig. 1 A). Blood skewing was strongly correlated with plaque skewing (P = 0.0001) (Fig. 1B). Age was not associated with blood (10-year β = − 0.14; P = 0.89, Additional file 1: Fig. S2 A, B) nor with plaque skewing (10-year β = 0.54; P = 0.43, Additional file 1: Fig. S2 C, D). The baseline characteristics showed no significant difference between the populations with skewed and non-skewed plaque (Table 1) or blood (Additional file 1: Table S1). The patients presenting skewed plaques were on average 67 years old and had a median BMI of 25, while the ones presenting with non-skewed plaques were on average 66 years old and had a median BMI of 26. Also, 37% of the skewed group had a history of PAOD and 29% of CAD while in the non-skewed group, 28% had history PAOD and 23% of CAD. Smokers were reported to be 41% in the skewed and 53% in the non-skewed group and the presence of diabetes was 18% and 19%, respectively.

To assess whether the extent of skewing (i.e., how skewed a plaque is) was important in relation to baseline characteristics, we created a categorical binned variable where X-skewing was classified in 4 levels (No, Low, Mid, High). Baseline characteristics of these 4 levels are shown in Additional file 1: Table S2.

### XCI skewing in different plaque phenotypes

To investigate whether XCI skewing in plaque was more common in a specific plaque phenotype, we assessed the relation between plaque skewing and histological plaque characteristics. For this, fat content (> 10% and > 40%), amount of calcification, amount of collagen, presence of plaque hemorrhage, macrophage, smooth muscle cell, neo-vessel and glycophorin content within the plaque (Table 2) was studied [28]. Univariate analysis showed a linear association between plaque skewing (Continuous variable) and plaque hemorrhage (OR: 1.44 [1.06–1.98]; P = 0.02), but not when using the dichotomous variable (Table 2). Data on the binned plaque skewing levels are showed in Additional file 1: Table S3. As plaque skewing showed a significant association with plaque hemorrhage, we examined the correlation between blood skewing and plaque hemorrhage using the XCI data from blood of 55 female patients. Our univariate analysis revealed that the confidence intervals were very wide, indicative of low power. Nevertheless, effect sizes were small suggesting that there is no linear association between blood skewing and plaque hemorrhage (OR: 1.10 [0.70–1.75], P = 0.67). Accordingly, the limited sample size renders our analysis underpowered, precluding the derivation of significant conclusions.

**Table 2:** Association of plaque skewing with plaque characteristics

	XCI skewing	Odds ratio [95% CI]	β [95% CI]	P value
Fat content (> 10%)	Dichotomous (Non-skewed)	(1.0) ref.	–	–
	Dichotomous (Skewed)	0.87 [0.45 to 1.71]	–	0.69
	*Continuously	0.96 [0.71 to 1.31]	–	0.82
Fat content (> 40%)	Dichotomous (Non-skewed)	(1.0) ref.	–	–
	Dichotomous (Skewed)	1.41 [0.64 to 3.22]	–	0.39
	*Continuously	1.08 [0.74 to 1.54]	–	0.67
Calcification (major)	Dichotomous (Non-skewed)	(1.0) ref.	–	–
	Dichotomous (Skewed)	1.37 [0.71 to 2.68]	–	0.35
	*Continuously	1.08 [0.80 to 1.48]	–	0.60
Collagen (major)	Dichotomous (Non-skewed)	(1.0) ref.	–	–
	Dichotomous (Skewed)	0.80 [0.34 to 1.88]	–	0.61
	*Continuously	0.96 [0.66 to 1.42]	–	0.82
Plaque Hemorrhage (major)	Dichotomous (Non-skewed)	(1.0) ref.	–	–
	Dichotomous (Skewed)	1.50 [0.79 to 2.87]	–	0.22
	*Continuously	1.44 [1.06 to 1.98]	–	0.02
Macrophage (major)	Dichotomous (Non-skewed)	(1.0) ref.	–	–
	Dichotomous (Skewed)	0.77 [0.41 to 1.45]	–	0.42
	*Continuously	1.03 [0.77 to 1.39]	–	0.80
Smooth muscle cells (major)	Dichotomous (Non-skewed)	(1.0) ref.	–	–
	Dichotomous (Skewed)	0.98 [0.46 to 2.08]	–	0.96
	*Continuously	0.92 [0.66 to 1.30]	–	0.66
**Neo-vessels (major)	Dichotomous (Non-skewed)	(1.0) ref.	–	–
	Dichotomous (Skewed)	0.88 [0.44 to 1.77]	–	0.72
	*Continuously	0.93 [0.68 to 1.28]	–	0.62
***Glycophorin (increase of plaque area)	Dichotomous (Non-skewed)	–	(1.0) ref.	–
	Dichotomous (Skewed)	–	0.09 [-0.26 to 0.45]	0.60
	*Continuously	–	0.02 [-0.13 to 0.18]	0.77

*Calculated for 10 points percentage of XCI skewing. Data transformed with bestNormalize package in R: **Binarize technique; ***The Ordered Quantile Normalization technique

In addition, in our pursuit to elucidate the potential mechanisms behind XCI skewing in plaques, we used RNA samples from 73 out of the 154 donors available from a previous study [30]. We conducted a differential gene expression analysis, to compare skewed and non-skewed plaques, correcting for plaque hemorrhage (Additional file 1: Fig. S3A). We listed the top 10 differentially expressed genes based on the nominal P-value (Additional file 1: Fig. S3B). However, upon adjusting for multiple testing, we found no statistically significant differences between the skewed and non-skewed plaques.

### Risk factors do not influence the relation between plaque skewing and plaque hemorrhage

Although risk factors did not show any significant differences between skewed and non-skewed plaques, we studied whether they modulate the association between plaque skewing and plaque hemorrhage (OR [95% CI]: 1.44 [1.06–1.98]; P = 0.02). We used classical cardiovascular risk factors, such as age, body mass index (BMI), smoking, diabetes mellitus and glomerular filtration rate to correct the model for one risk factor at the time. We found that BMI slightly attenuated the association of plaque skewing with plaque hemorrhage (OR [95% CI]: 1.37 [0.99–1.94]; P = 0.07). Also, smoking marginally increased this association (OR [95% CI]: 1.50 [1.10–2.09]; P = 0.01) (Table 3). Additional file 1: Table S4 shows the data on the binned plaque skewing levels.

**Table 3:** Effect of classical cardiovascular risk factors on the association between plaque skewing and plaque hemorrhage

XCI skewing	Plaque hemorrhage (major)	
	Odds ratio [95% CI]	P value
Dichotomous (Non-skewed)	(1.0) ref.	–
Dichotomous (Skewed)	1.50 [0.79 to 2.87]	0.22
Continuously*	1.44 [1.06 to 1.98]	0.02
Adjusted for: Age		
Dichotomous (Non-skewed)	(1.0) ref.	–
Dichotomous (Skewed)	1.49 [0.78 to 2.86]	0.22
Continuously*	1.43 [1.06 to 1.98]	0.02
Adjusted for: BMI		
Dichotomous (Non-skewed)	(1.0) ref.	–
Dichotomous (Skewed)	1.49 [0.76 to 2.97]	0.25
Continuously*	1.37 [0.99 to 1.94]	0.07
Adjusted for: Smoking		
Dichotomous (Non-skewed)	(1.0) ref.	–
Dichotomous (Skewed)	1.62 [0.84 to 3.14]	0.15
Continuously*	1.50 [1.10 to 2.09]	0.01
Adjusted for: Diabetes Mellitus		
Dichotomous (Non-skewed)	(1.0) ref.	–
Dichotomous (Skewed)	1.50 [0.79 to 2.87]	0.22
Continuously*	1.44 [1.06 to 1.98]	0.02
Adjusted for: GFR (MDRD)		
Dichotomous (Non-skewed)	(1.0) ref.	–
Dichotomous (Skewed)	1.46 [0.77 to 2.80]	0.25
Continuously*	1.44 [1.06 to 1.99]	0.02
Adjusted for: BMI + Smoking		
Dichotomous (Non-skewed)	(1.0) ref.	–
Dichotomous (Skewed)	1.53 [0.77 to 3.05]	0.23
Continuously*	1.38 [0.99 to 1.96]	0.06

*Calculated for 10 points percentage of XCI skewing

### Association with cardiovascular endpoints

**Fig. 2:**
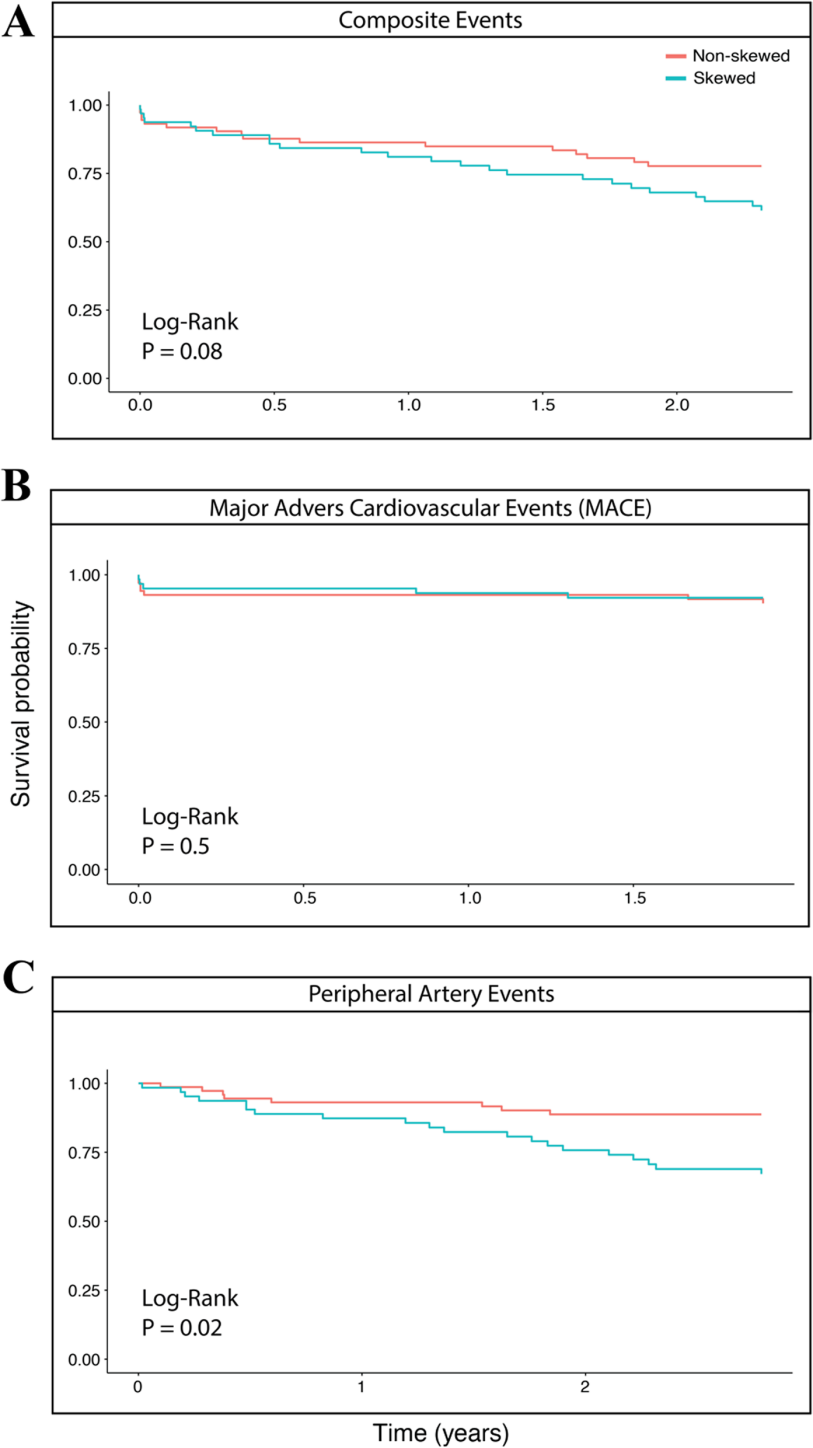
XCI plaque skewing (yes/no), cox proportional hazards models for: **A** composite event-free survival (P = 0.08); **B** major event-free survival (P = 0.5); **C** peripheral artery event-free survival (P = 0.02). Models adjusted for BMI and current smoking. The survival probability is predicted until the occurrence of the last event

We investigated if XCI skewing in plaque was associated with the incidence of secondary cardiovascular events in a 3-year follow-up after endarterectomy. For this, we used survival analysis, Kaplan–Meier (Additional file 1: Fig. S4) and cox proportional hazard model, corrected for BMI and current smoking (Fig. 2), to assess the association of plaque skewing with composite (41 events, 27%), major adverse cardiovascular (MACE; 13 events, 9%) and peripheral artery events (28 events, 19%) during 3-years follow-up.

Women with skewed plaques had no more MACE (Fig. 2B) compared to women without skewing (HR [95%CI]: 0.89 [0.30–2.65]; P = 0.84), also when correcting for BMI and current smoking (HR [95%CI]: 0.80 [0.25–2.53]; P = 0.70) (Table 4). During 3 years of follow-up, plaque skewing was also not significantly associated with composite endpoints (Table 4; Fig. 2 A). However, a trend was observed in the association of dichotomous plaque skewing with composite events (HR [95%CI]: 1.70 [0.91–3.19]; P = 0.09) which increased slightly in strength when correcting the model for BMI and current smoking (HR [95%CI]: 1.82 [0.96–3.45]; P = 0.07). We found that women with skewed plaques had significantly more peripheral artery events during 3 years follow up (Table 4; Fig. 2C) when using both continuous (HR [95%CI]: 3.14 [1.38–7.18]; P = 0.01) and dichotomous XCI (HR [95%CI]: 1.46 [1.09–1.97]; P = 0.007) in multivariate analyses. Also adjusting for plaque hemorrhage did not change the relation between XCI and outcome (data not shown). We performed similar analysis using the binned plaque skewing levels which are showed in Additional file 1: Table S5, Fig. S5 and Fig. S6.

**Table 4:** Association of plaque skewing with secondary cardiovascular endpoints during 3-years follow-up

XCI skewing	Crude analysis		Adjusted for BMI and Smoking	
	Hazard ratio [95% CI]	P value	Hazard ratio [95% CI]	P value
Composite event				
Dichotomous (Non-skewed)	(1.0) ref.	–	(1.0) ref.	–
Dichotomous (Skewed)	1.70 [0.91 to 3.19]	0.1	1.82 [0.96 to 3.45]	0.07
Continuously*	1.24 [0.96 to 1.62]	0.1	1.21 [0.93 to 1.57]	0.15
Major cardiovascular event (MACE)				
Dichotomous (Non-skewed)	(1.0) ref.	–	(1.0) ref.	–
Dichotomous (Skewed)	0.89 [0.30 to 2.65]	0.84	0.80 [0.25 to 2.53]	0.7
Continuously*	0.98 [0.59 to 1.62]	0.94	0.81 [0.47 to 1.41]	0.46
Peripheral artery event				
Dichotomous (Non-skewed)	(1.0) ref.	–	(1.0) ref.	–
Dichotomous (Skewed)	2.85 [1.25 to 6.47]	0.012	3.14 [1.38 to 7.18]	0.007
Continuously*	1.45 [1.07 to 1.97]	0.018	1.46 [1.09 to 1.97]	0.011

*Calculated for 10 points percentage of XCI skewing

## Discussion

In our study, we investigated XCI status of atherosclerotic plaques by examining the DNA methylation status of the polymorphic AR locus (HUMARA assay) and studied the occurrence of XCI skewing in carotid plaques on the DNA of 154 female atherosclerotic patients and its association with cardiovascular risk factors, histological and clinical outcomes.

We report that XCI skewing in plaques was present in half (49.6%) of the population. XCI plaque skewing was not associated with cardiovascular risk factors, but it was strongly associated with the presence of plaque hemorrhage. Plaque hemorrhage has been widely studied in the atherosclerosis field, and known to be a common phenomenon in advanced atherosclerotic plaques [4, 31]. In carotid lesions, it was found associated with an increased risk of secondary cardiovascular events in men but not in women [4]. Also, in our study, adjusting for plaque hemorrhage in the multivariate analysis did not change the relation between XCI and incident peripheral artery events. This may be explained by different mechanisms underlying symptomatic plaques in women and men [32]. In female plaques, cell plasticity seems to be dominant pointing to plaque erosion as a symptomatic mechanism in women as compared to plaque rupture in male plaques. We studied the plaque skewing and plaque hemorrhage further and hypothesized that infiltration of erythrocytes from either the bloodstream or leaky neo-vessels within the plaque [31] might be the driver of the association between plaque skewing and plaque hemorrhage in these female plaques. However, we did not find an association with glycophorin which is an erythrocyte-specific protein used to identify plaque bleeding [33]. A possible explanation for the lack of this association may be found in the quantification method. Indeed, plaque hemorrhage includes an expert assessment of the bleeding in the plaque while glycophorin staining has been measured via a computed process that cannot be subjected to the user experience [33, 34]. Hence, the associations with plaque hemorrhage or glycophorin data can be considered independent because none of them fully represents bleeding in atherosclerotic plaques. We replicated the same association analysis using the binned plaque skewing variable and we confirmed the association between plaque skewing and plaque hemorrhage also finding a dose-depended relationship.

We also found that age was not associated with plaque skewing although several studies showed that age was dose-dependently associated with skewing in general population [35–37]. This may be explained by the differences in the population studied, the tissue analyzed, and/or the assay used. We studied the XCI status of end-stage carotid plaques obtained from symptomatic women that have limited age distribution which may explain the lack of association. Also, the lack of healthy individuals in our study limits us to comment of the whether the prevalence of skewing in plaque is disease-related.

One of the possible mechanisms to explain XCI skewing in atherosclerotic lesions is the clonal expansion of cells involved in plaque formation [38]. Studies conducted in mice showed that macrophages undergo polyclonal expansion within atherosclerotic plaques, indicating that the proliferation of macrophages is already present in the plaque [39, 40]. However, in our study, we did not identify differences in the content of macrophages associated with skewed XCI. Potentially this could be due to the differences between mice and human plaques and/or the fact that the plaques in our study are in an advanced stage compared to plaques studied in mice.

Besides macrophages, the clonal expansion of smooth muscle cells was also proposed as a potential mechanism for XCI skewing in human carotid plaques, resulting in a population of monoclonal cells with the same XCI pattern [41, 42]. So, if the proposed hypothesis was true, a skewed XCI pattern would lead to a SMC-rich stable/fibrotic atherosclerotic plaque phenotype. However, this was not observed in our analysis arguing against clonal expansion being the driver of XCI. On the other hand, we observed a relation with peripheral artery event which are characterized by fibrous lesions, known to contain a large number of smooth muscle cells with enhanced proliferation capacities. This proliferative activity of smooth muscle cells seems to differ between vascular beds and in peripheral artery has a role in stabilizing of those lesions [42]. These SMC-mechanisms may be explained by clonal expansion which takes place in peripheral arteries, leading to events affecting limb arteries. In our population, we were unable to identify a higher content of smooth muscle cells associated with skewed XCI. One of the possible explanations for this may be due to the advanced disease stage on the patients in our study. It may be that the XCI skewing in carotid plaques plays a role on the cellular composition of plaques in an earlier phase of the disease and initially affects the plaque to become fibrotic. As this population is female only, and thereby the prevalence of fibrous plaques is already high, we may not find an association due to the lack of variation in smooth muscle cell content.

We also assessed the association between plaque skewing and secondary cardiovascular events to assess the clinical relevance of the presence of skewing. In our study we did not find an association of plaque skewing with either MACE or composite endpoints. Interestingly, in a secondary analysis we found that skewed plaques were associated with a higher occurrence of peripheral artery events during a 3-year follow-up period. Indeed, it is known that plaques found in the ilio-femoral arteries are often more fibrous than carotid plaques [43, 44], this in combination with our results may suggest XCI may be related to fibrotic processes in the periphery rather than the carotid arteries. However, peripheral artery endpoint was not the primary focus of the study and therefore this observation can only be seen as a sub-analysis which merits careful consideration and should be validated in an independent study.

This study had some limitations, the HUMARA assay may be influenced by preferential amplification of AR alleles with shorter repeats [45]. Also, the expression of a single X-linked locus may not reflect the expression status of the entire X-chromosome as there are genes with variable levels of escape from X-inactivation in the healthy population. Another limitation is the lack of healthy control tissues, which were previously reported to present extensive XCI skewing in a population of healthy women [19]. In our study, we did not examine whether XCI skewing is more prevalent in diseased arteries compared to normal ones. This might be challenging to study as atherosclerosis is present in the arteries of women without apparent arterial disease. Nevertheless, we observed a high occurrence of XCI skewing in blood samples (67% of the samples). This aligns with previous reports where the prevalence of XCI skewing is around 70% in individuals older than 55 years [16]. A possible explanation for the observed higher XCI skewing in blood compared to plaques may lie in the cellular composition of these tissues. Plaques consist of a complex mixture of cellular and acellular components, with a prominent presence of mesenchymal cells [46, 47]. Conversely, blood is characterized by a high abundance of leukocytes [48] that commonly undergo clonal proliferation, known to occur more frequently with advancing age [49]. This variation in cell composition likely contributes to the observed differences in XCI skewing prevalence between these tissue types. However, it is important to note that the mechanisms underlying XCI skewing are complex and multifactorial, and further research is needed to understand the relationship between tissue composition and XCI skewing.

Additionally, the major limitation of our study is the lack of power given by the limited number of patients included together with the fact that they only represent a snapshot of end-stage carotid plaques. However, this exploratory study highlights the need for further studies on the role of XCI skewing in atherosclerosis.

In conclusion, XCI skewing is common in carotid plaques of females and is predictive for the occurrence of peripheral artery events within 3 years after carotid endarterectomy.

### Perspective and significance

Despite the well-known differences in the behavior of plaques between males and females, with female plaques being perceived as more stable and fibrous [2–4], the knowledge on XCI has been largely unexplored. Our study sheds light on the prevalence of X chromosome inactivation (XCI) skewing in female atherosclerotic plaques and its association with cardiovascular risk factors, histological plaque data, and clinical data. We found that XCI skewing was common in carotid plaques of females, with 49% of the plaques exhibiting skewing. None of the clinical risk factors were associated with plaque skewing. Furthermore, we found that skewed plaques were specifically associated with peripheral artery events within 3 years after carotid endarterectomy. These findings highlight the significance of understanding XCI skewing in female atherosclerotic plaques and its potential as a predictor of cardiovascular events. This study sheds light on the importance of sex-specific mechanisms in atherosclerosis.

## Supplementary Information

**Additional file 1.** Supplementary Data.

## Data Availability

All data generated or analyzed during this study are included in this article.

## Abbreviations

BMI: Body mass index
CAD: Coronary artery disease
CEA: Carotid endarterectomy
HUMARA: Human androgen receptor assay
MACE: Major adverse cardiovascular events
PAOD: Peripheral artery occlusive disease
SMC: Smooth muscle cell
XCI: X chromosome inactivation
