# Supplementary material for: X chromosome inactivation skewing is common in advanced carotid atherosclerotic lesions in females and predicts secondary peripheral artery events
Source: Biol Sex Differ. 2023 Jul 5;14:43. doi: 10.1186/s13293-023-00527-6 (PMC10324263; doi:10.1186/s13293-023-00527-6)
Supplement: Supplementary file 1 — Additional file 1. Supplementary Data. [file 13293_2023_527_MOESM1_ESM.docx]

**Table S1. Baseline characteristics of patients with and without XCI skewing in blood.**

| XCI Skewing: | Overall | No Skewed | Skewed | P Value |
| --- | --- | --- | --- | --- |
| N (%) | 55 | 18 | 37 | - |
| Age in years (mean, SD) | 66 (10) | 66 (11) | 66 (9) | 0.75 |
| BMI (median, IQR) | 27 [24, 30] | 27 [24, 28] | 27 [25, 31] | 0.29 |
| Current Smoker, yes (%) | 22 (42) | 6 (35) | 16 (44) | 0.74 |
| Diabetes Mellitus, yes (%) | 13 (24) | 2 (11) | 11 (30) | 0.24 |
| Hypertension, yes (%) | 48 (87) | 16 (89) | 32 (86) | 1.00 |
| Hypercholesterolemia, yes (%) | 35 (67) | 12 (67) | 23 (68) | 1.00 |
| History of coronary artery disease (%) | 18 (33) | 7 (39) | 11 (30) | 0.71 |
| History of PAOD (%) | 12 (22) | 4 (22) | 8 (22) | 1.00 |
| Use of antiplatelet therapy (%) | 52 (95) | 15 (83) | 37 (100) | 0.05 |
| Use of lipid-lowering drugs (%) | 42 (76) | 12 (67) | 30 (81) | 0.40 |
| GFR (MDRD) mL/min per 1.73 m2 (mean, SD) | 74 (19) | 70 (19) | 75 (19) | 0.36 |
| LDL in mg/dL (median, IQR) | 102 [77, 136] | 113 [90, 153] | 97 [74, 133] | 0.29 |
| HDL in mg/dL (median, IQR) | 44 [37, 51] | 46 [41, 49] | 44 [35, 51] | 0.45 |
| Total cholesterol in mg/dL (median, IQR) | 180 [145, 219] | 183 [150, 230] | 158 [144, 217] | 0.49 |
| Triglyceride levels in mg/dL (median, IQR) | 131 [104, 188] | 130 [104, 157] | 138 [101, 195] | 0.53 |
| Presenting symptoms (%) | | | | 0.11 |
| Asymptomatic | 13 (24) | 7 (39) | 6 (17) |  |
| Transient Ischemic Attack (TIA) | 28 (52) | 9 (50) | 19 (53) |  |
| Stroke | 13 (24) | 2 (11) | 11 (31) |  |

**Table S2. Baseline characteristics of patients with binned XCI skewing in plaque.**

| XCI Skewing: | No | Low | Mid | High | P Value |
| --- | --- | --- | --- | --- | --- |
| N (%) | 57 (37) | 46 (30) | 28 (18) | 23 (15) | - |
| Age in years (mean, SD) | 65 (9) | 67 (10) | 68 (9) | 65 (8) | 0.49 |
| BMI (median, IQR) | 26 [23, 28] | 25 [23, 28] | 25 [23, 29] | 27 [23, 29] | 0.57 |
| Current Smoker, yes (%) | 28 (50) | 24 (53) | 10 (36) | 9 (41) | 0.44 |
| Diabetes Mellitus, yes (%) | 12 (21) | 5 (11) | 4 (14) | 8 (35) | 0.10 |
| Hypertension, yes (%) | 52 (91) | 39 (85) | 25 (89) | 19 (83) | 0.65 |
| Hypercholesterolemia, yes (%) | 38 (69) | 30 (65) | 21 (78) | 16 (70) | 0.73 |
| History of coronary artery disease (%) | 13 (23) | 8 (17) | 14 (50) | 5 (22) | 0.01 |
| History of PAOD (%) | 16 (28) | 15 (33) | 11 (39) | 8 (35) | 0.76 |
| Use of antiplatelet therapy (%) | 50 (88) | 46 (100) | 26 (93) | 22 (96) | 0.09 |
| Use of lipid-lowering drugs (%) | 43 (75) | 35 (76) | 23 (82) | 18 (78) | 0.91 |
| GFR (MDRD) mL/min per 1.73 m2 (mean, SD) | 68 (21) | 67 (18) | 72 (18) | 78 (18) | 0.14 |
| LDL in mg/dL (median, IQR) | 106 [74, 145] | 105 [81, 144] | 119 [90, 153] | 110 [85, 135] | 0.95 |
| HDL in mg/dL (median, IQR) | 46 [37, 55] | 46 [36, 59] | 44 [40, 53] | 47 [37, 58] | 1.00 |
| Total cholesterol in mg/dL (median, IQR) | 185 [147, 227] | 196 [149, 231] | 203 [169, 234] | 191 [145, 217] | 0.89 |
| Triglyceride levels in mg/dL (median, IQR) | 119 [97, 179] | 152 [100, 220] | 113 [96, 150] | 142 [81, 172] | 0.72 |
| Presenting symptoms (%) | | | | | 0.44 |
| Asymptomatic | 10 (19) | 10 (25) | 4 (18) | 1 (5) |  |
| TIA | 27 (50) | 23 (57) | 13 (59) | 14 (70) |  |
| Stroke | 17 (31) | 7 (18) | 5 (23) | 5 (25) |  |

XCI Skewing: **No** = skewing percentage <60%; **Low** = skewing percentage ≥60% and <70%; **Mid** = skewing percentage as ≥70% and <80%; **High** = skewing percentage ≥80%. BMI: body mass index; GFR: glomerular filtration rate; HDL: high-density lipoprotein; IQR: interquartile range; LDL: low density lipoprotein; MDRD, modification of diet in renal disease; PAOD: peripheral arterial occlusive disease; TIA: transient ischemic attack; XCI: X chromosome inactivation.

**Table S3**. **Association of binned plaque skewing with plaque characteristics.**

|  | Univariate | | Multivariate: adjusted for CAD History | |
| --- | --- | --- | --- | --- |
| XCI Skewing | **Odds Ratio [95% CI]** | **P Value** | **Odds Ratio [95% CI]** | **P Value** |
|  | **Atheroma size (>10%)** | | | |
| No | 1.0 (ref) | - | 1.0 (ref) | - |
| Low | 1.08 [0.47 to 2.54] | 0.85 | 1.06 [0.46 to 2.50] | 0.89 |
| Mid | 0.63 [0.25 to 1.62] | 0.33 | 0.72 [0.27 to 1.92] | 0.51 |
| High | 0.89 [0.32 to 2.55] | 0.82 | 0.89 [0.32 to 2.57] | 0.82 |
|  | **Atheroma size (>40%)** | | | |
| No | 1.0 (ref) | - | 1.0 (ref) | - |
| Low | 1.84 [0.70 to 4.99] | 0.22 | 1.81 [0.69 to 4.92] | 0.23 |
| Mid | 1.13 [0.32 to 3.68] | 0.84 | 1.35 [0.37 to 4.57] | 0.64 |
| High | 1.10 [0.27 to 3.83] | 0.89 | 1.10 [0.27 to 3.86] | 0.88 |
|  | **Calcification (major)** | | | |
| No | 1.0 (ref) | - | 1.0 (ref) | - |
| Low | 3.18 [1.38 to 7.72] | 0.008 | 3.26 [1.41 to 7.96] | 0.007 |
| Mid | 3.00 [1.14 to 8.65] | 0.03 | 2.67 [0.98 to 7.84] | 0.06 |
| High | 1.30 [0.49 to 3.52] | 0.59 | 1.30 [0.49 to 3.53] | 0.60 |
|  | **Collagen (major)** | | | |
| No | 1.0 (ref) | - | 1.0 (ref) | - |
| Low | 0.58 [0.20 to 1.63] | 0.30 | 0.59 [0.21 to 1.66] | 0.32 |
| Mid | 1.00 [0.28 to 4.05] | 1.00 | 0.91 [0.25 to 3.80] | 0.89 |
| High | 0.79 [0.22 to 3.25] | 0.73 | 0.79 [0.22 to 3.25] | 0.73 |
|  | **Plaque Hemorrhage (major)** | | | |
| No | 1.0 (ref) | - | 1.0 (ref) | - |
| Low | 1.01 [0.46 to 2.22] | 0.97 | 1.01 [0.46 to 2.22] | 0.97 |
| Mid | 1.24 [0.50 to 3.14] | 0.64 | 1.26 [0.49 to 3.27] | 0.63 |
| High | 3.35 [1.15 to 11.29] | 0.03 | 3.35 [1.15 to 11.29] | 0.03 |
|  | **Macrophage (major)** | | | |
| No | 1.0 (ref) | - | 1.0 (ref) | - |
| Low | 1.30 [0.59 to 2.86] | 0.51 | 1.29 [0.59 to 2.84] | 0.53 |
| Mid | 0.59 [0.22 to 1.49] | 0.27 | 0.64 [0.24 to 1.66] | 0.36 |
| High | 1.55 [0.58 to 4.29] | 0.38 | 1.56 [0.58 to 4.30] | 0.38 |
|  | **Smooth Muscle Cells (major)** | | | |
| No | 1.0 (ref) | - | 1.0 (ref) | - |
| Low | 0.87 [0.34 to 2.22] | 0.76 | 0.86 [0.33 to 2.20] | 0.75 |
| Mid | 0.68 [0.24 to 1.98] | 0.47 | 0.74 [0.25 to 2.22] | 0.58 |
| High | 0.98 [0.31 to 3.45] | 0.98 | 0.98 [0.31 to 3.45] | 0.98 |
|  | **Neo-vessels (major)**** | | | |
| No | 1.0 (ref) | - | 1.0 (ref) | - |
| Low | 0.48 [0.19 to 1.14] | 0.10 | 0.46 [0.19 to 1.12] | 0.09 |
| Mid | 0.37 [0.13 to 1.04] | 0.06 | 0.43 [0.14 to 1.23] | 0.12 |
| High | 0.86 [0.30 to 2.52] | 0.78 | 0.83 [0.28 to 2.47] | 0.74 |
|  | **Glycophorin (increase of plaque area)***** | | | |
|  | **β [95% CI]** | **P Value** | **β [95% CI]** | **P Value** |
| No | 1.0 (ref) | - | 1.0 (ref) | - |
| Low | 0.39 [-0.04 to 0.83] | 0.08 | 0.40 [-0.04 to 0.84] | 0.07 |
| Mid | -0.10 [-0.61 to 0.42] | 0.71 | -0.13 [-0.66 to 0.40] | 0.63 |
| High | 0.24 [-0.28 to 0.77] | 0.36 | 0.25 [-0.28 to 0.78] | 0.35 |

*Calculated for 10 points percentage of XCI skewing. Data transformed with bestNormalize package in R: **Binarize technique; *** The Ordered Quantile Normalization technique.

**Table S4. Effect of classical cardiovascular risk factors on the association between binned plaque skewing levels and plaque hemorrhage.**

|  | *Plaque Hemorrhage (major) | |
| --- | --- | --- |
| XCI Skewing | **Odds Ratio [95% CI]** | **P Value** |
| No | 1.0 (ref) | - |
| Low | 1.01 [0.46 to 2.22] | 0.97 |
| Mid | 1.26 [0.49 to 3.27] | 0.63 |
| High | 3.35 [1.15 to 11.29] | 0.03 |
|  | Adjusted for: **Age** | |
| No | 1.0 (ref) | - |
| Low | 1.00 [0.46 to 2.20] | 0.99 |
| Mid | 1.24 [0.48 to 3.23] | 0.66 |
| High | 3.35 [1.16 to 11.31] | 0.03 |
|  | Adjusted for: **BMI** | |
| No | 1.0 (ref) | - |
| Low | 0.86 [0.37 to 1.94] | 0.71 |
| Mid | 1.13 [0.42 to 3.11] | 0.80 |
| High | 2.78 [0.85 to 10.96] | 0.11 |
|  | Adjusted for: **Smoking** | |
| No | 1.0 (ref) | - |
| Low | 1.00 [0.46 to 2.22] | 0.99 |
| Mid | 1.32 [0.51 to 3.48] | 0.56 |
| High | 4.33 [1.40 to 16.51] | 0.02 |
|  | Adjusted for: **Diabetes Mellitus** | |
| No | 1.0 (ref) | - |
| Low | 1.00 [0.46 to 2.22] | 0.99 |
| Mid | 1.25 [0.49 to 3.26] | 0.64 |
| High | 3.38 [1.16 to 11.47] | 0.03 |
|  | Adjusted for: **GFR (MDRD)** | |
| No | 1.0 (ref) | - |
| Low | 1.05 [0.48 to 2.32] | 0.90 |
| Mid | 1.28 [0.50 to 3.35] | 0.60 |
| High | 3.47 [1.18 to 11.82] | 0.03 |
|  | Adjusted for: **BMI + Smoking** | |
| No | 1.0 (ref) | - |
| Low | 0.88 [0.38 to 2.01] | 0.76 |
| Mid | 1.18 [0.43 to 3.28] | 0.74 |
| High | 2.89 [0.88 to 11.42] | 0.10 |

*Model corrected for CAD history.

**Table S5. Association of plaque skewing levels (binned variable) with secondary cardiovascular endpoints during 3-years follow-up.**

|  | Crude analysis | | Adjusted for CAD history, BMI and Smoking | |
| --- | --- | --- | --- | --- |
| XCI Skewing | **Hazard Ratio [95% CI]** | **P Value** | **Hazard Ratio [95% CI]** | **P Value** |
|  | **Composite Event** | | | |
| No | 1.0 (ref) | - | 1.0 (ref) | - |
| Low | 1.08 [0.48 to 2.42] | 0.84 | 1.10 [0.49 to 2.48] | 0.82 |
| Mid | 1.70 [0.75 to 3.90] | 0.20 | 1.78 [0.76 to 4.19] | 0.18 |
| High | 1.41 [0.56 to 3.55] | 0.46 | 1.37 [0.52 to 3.61] | 0.53 |
|  | **Major Cardiovascular Event (MACE)** | | | |
| No | 1.0 (ref) | - | 1.0 (ref) | - |
| Low | 0.64 [0.16 to 2.56] | 0.53 | 0.77 [0.19 to 3.21] | 0.72 |
| Mid | 1.06 [0.27 to 4.25] | 0.93 | 1.19 [0.26 to 5.32] | 0.83 |
| High | 0.43 [0.05 to 3.56] | 0.43 | 0.00 [0.00 to 0.00]* | 1.00* |
|  | **Peripheral Artery Event** | | | |
| No | 1.0 (ref) | - | 1.0 (ref) | - |
| Low | 1.71 [0.59 to 4.92] | 0.32 | 1.64 [0.56 to 4.79] | 0.36 |
| Mid | 3.14 [1.09 to 9.07] | 0.03 | 2.92 [0.98 to 8.74] | 0.05 |
| High | 2.87 [0.92 to 8.90] | 0.07 | 3.34 [1.07 to 10.36] | 0.04 |

*Due to lack of events, adjusted model for MACE for high plaque skewing was not possible to calculate


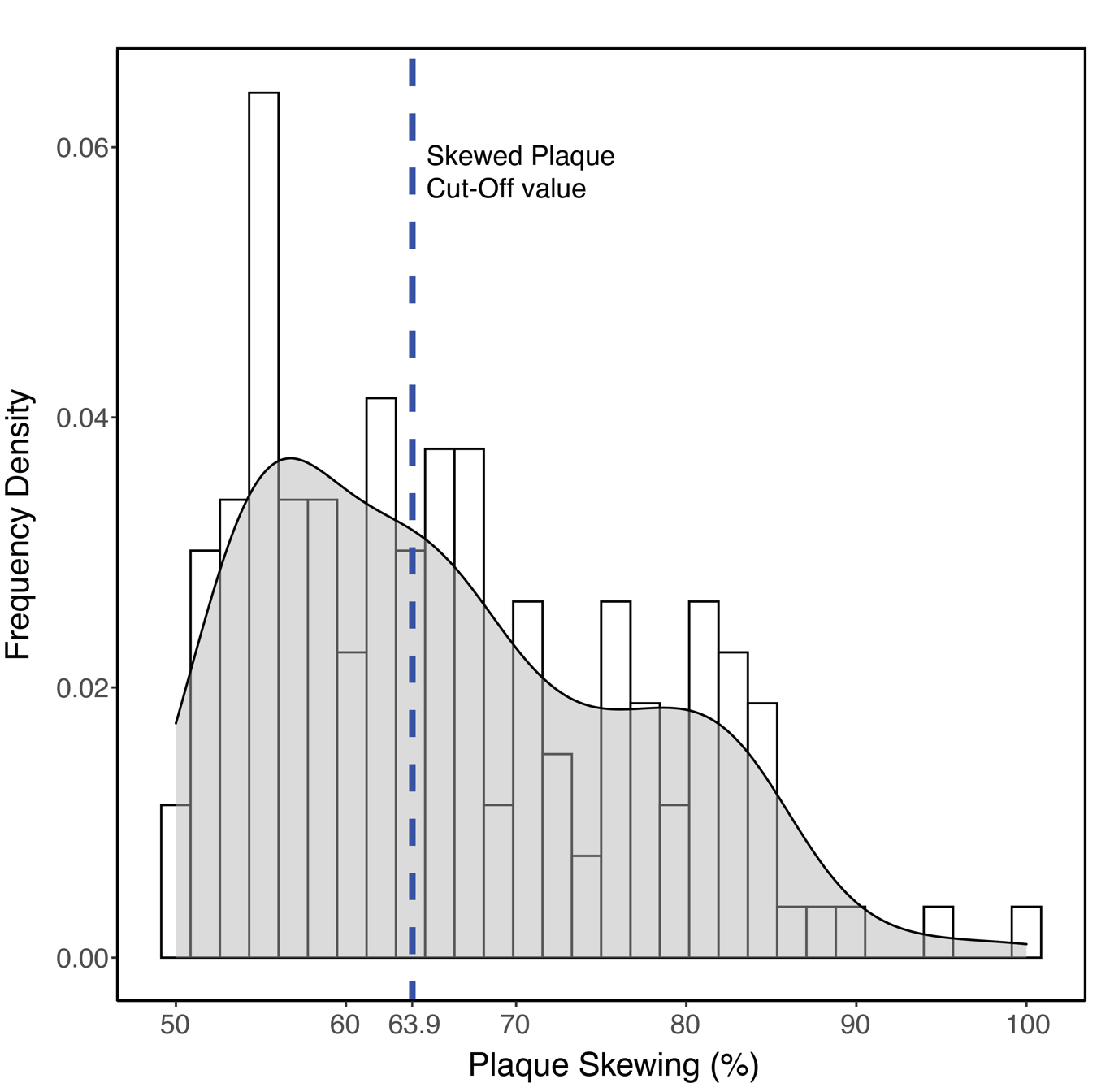


**Figure S1.** Determination of XCI skewing cut-off.


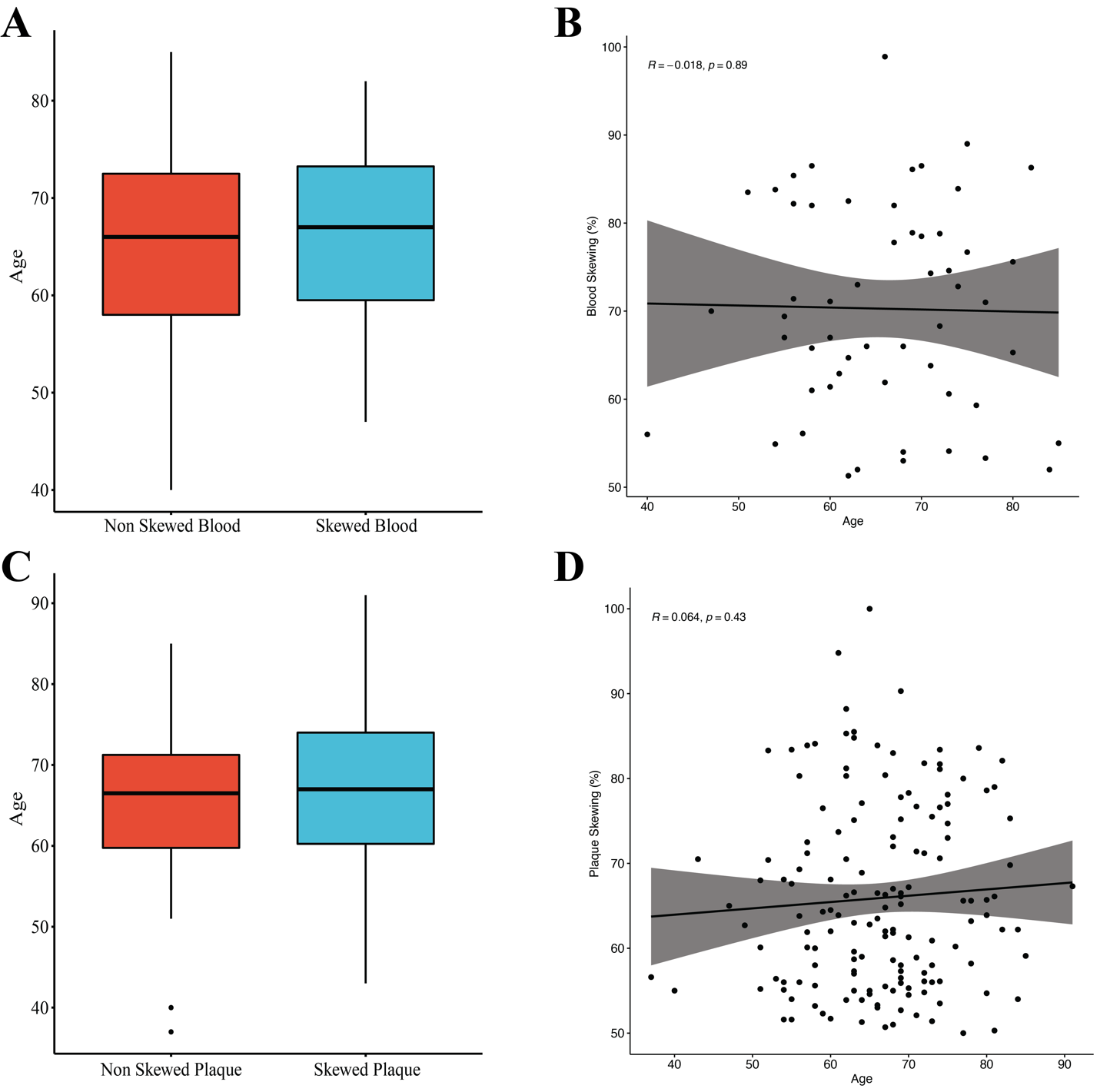


**Figure S2. Association of dichotomous XCI skewing and age. (A)** Bar plot showing the no differences in the age of skewed and non-skewed blood samples. **(B)** Scatter plot showing no association between blood skewing and age (β = -0.14/10 y; P = 0.89). **(C)** Bar plot showing the no differences in the age of skewed and non-skewed plaque samples. **(D)** Scatter plot showing no association between blood skewing and age (β = 0.54/10 y; P = 0.43)


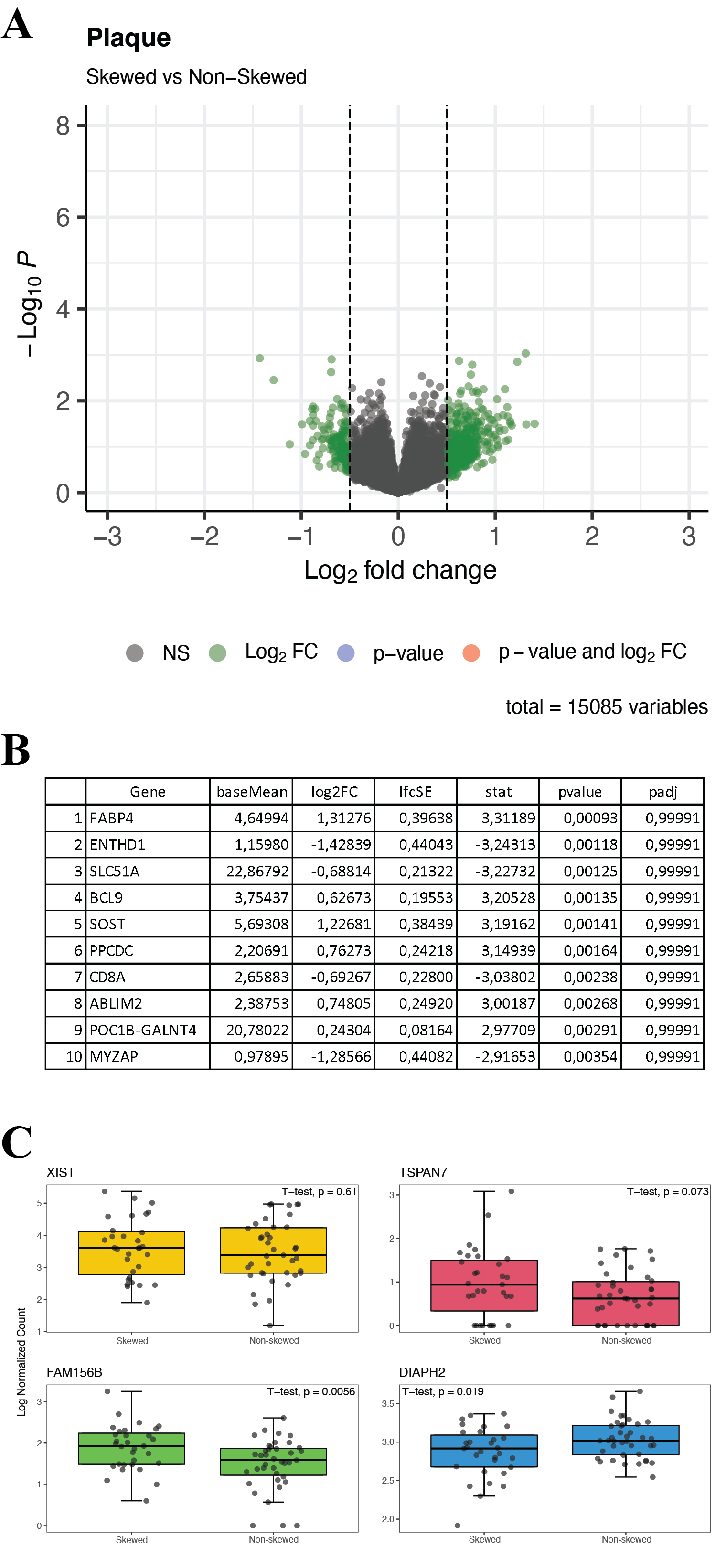


**Figure S3. Gene expression data of plaque samples**. (A) Volcano Plot showing differentially expressed gene in skewed versus non-skewed plaques. (B) Top 10 differentially expressed genes based on the nominal P-value.


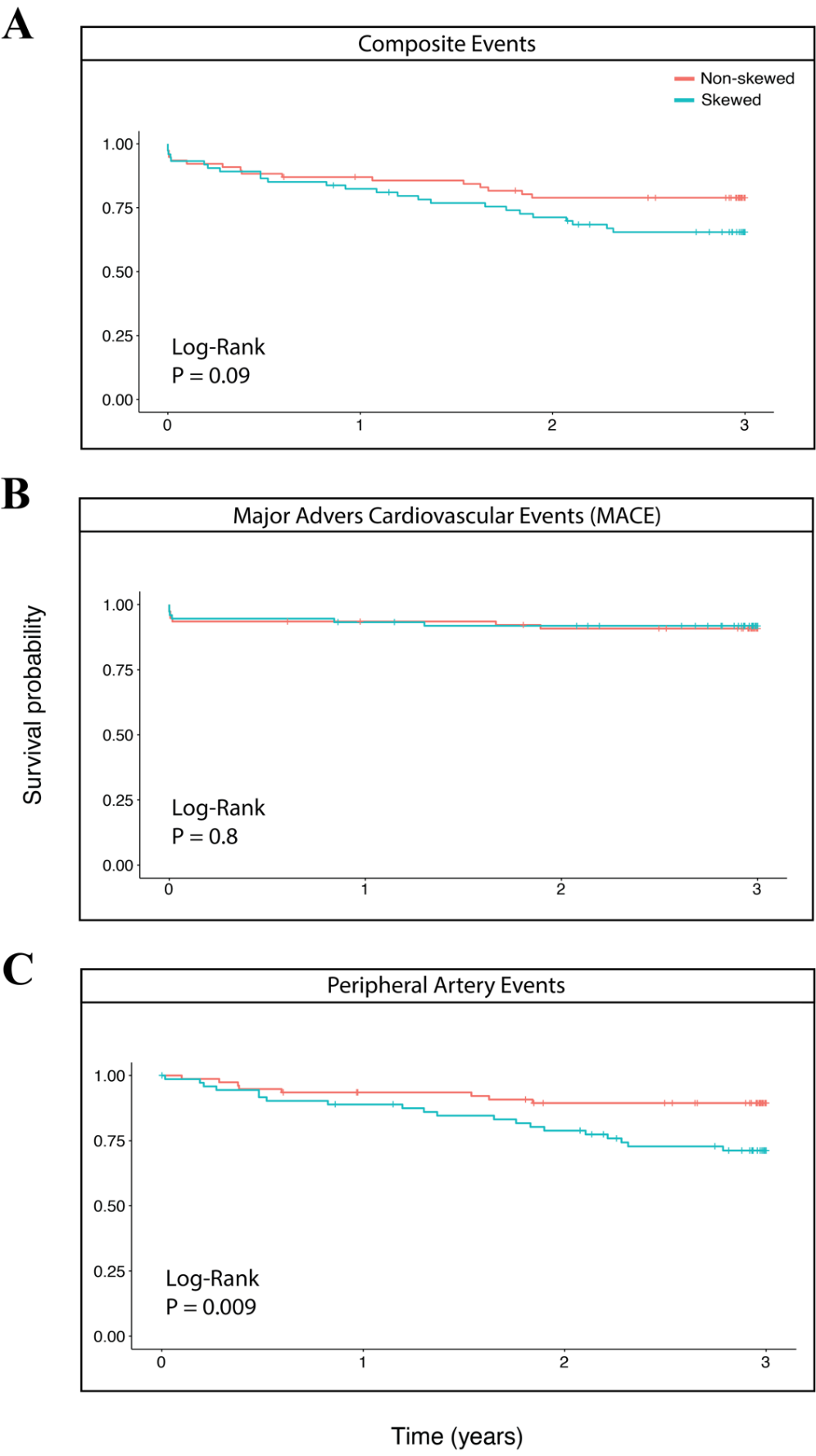


**Figure S4**. XCI plaque skewing (yes/no), Kaplan-Meyer curves for: (A) composite event-free survival (P = 0.09); (B) major event-free survival (P = 0.8); (C) peripheral artery event-free survival (P = 0.009).


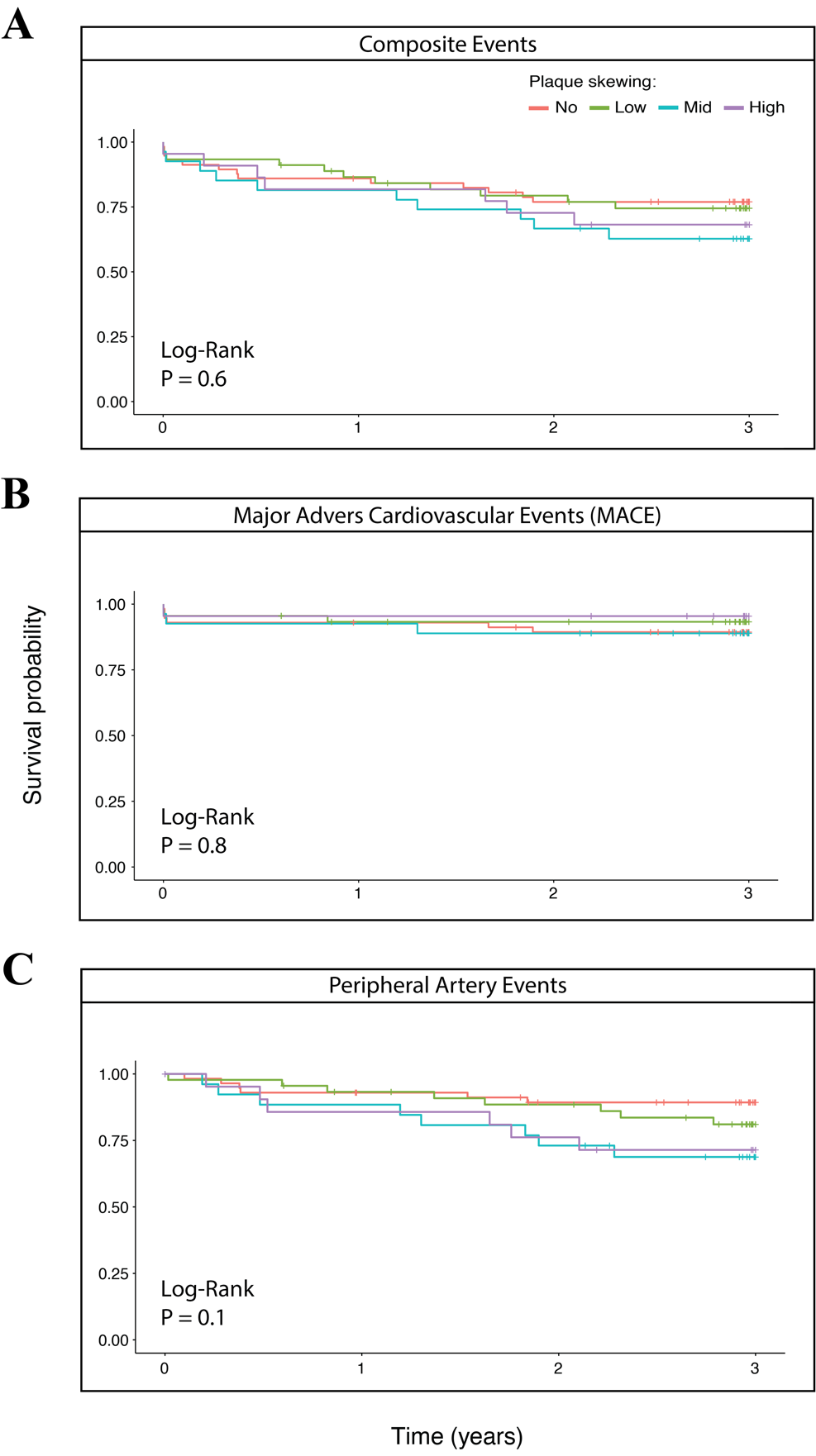


**Figure S5.** Binned plaque skewing levels, Kaplan-Meyer curves for: (A) composite event-free survival (P = 0.6); (B) major event-free survival (P = 0.8); (C) peripheral artery event-free survival (P = 0.1).


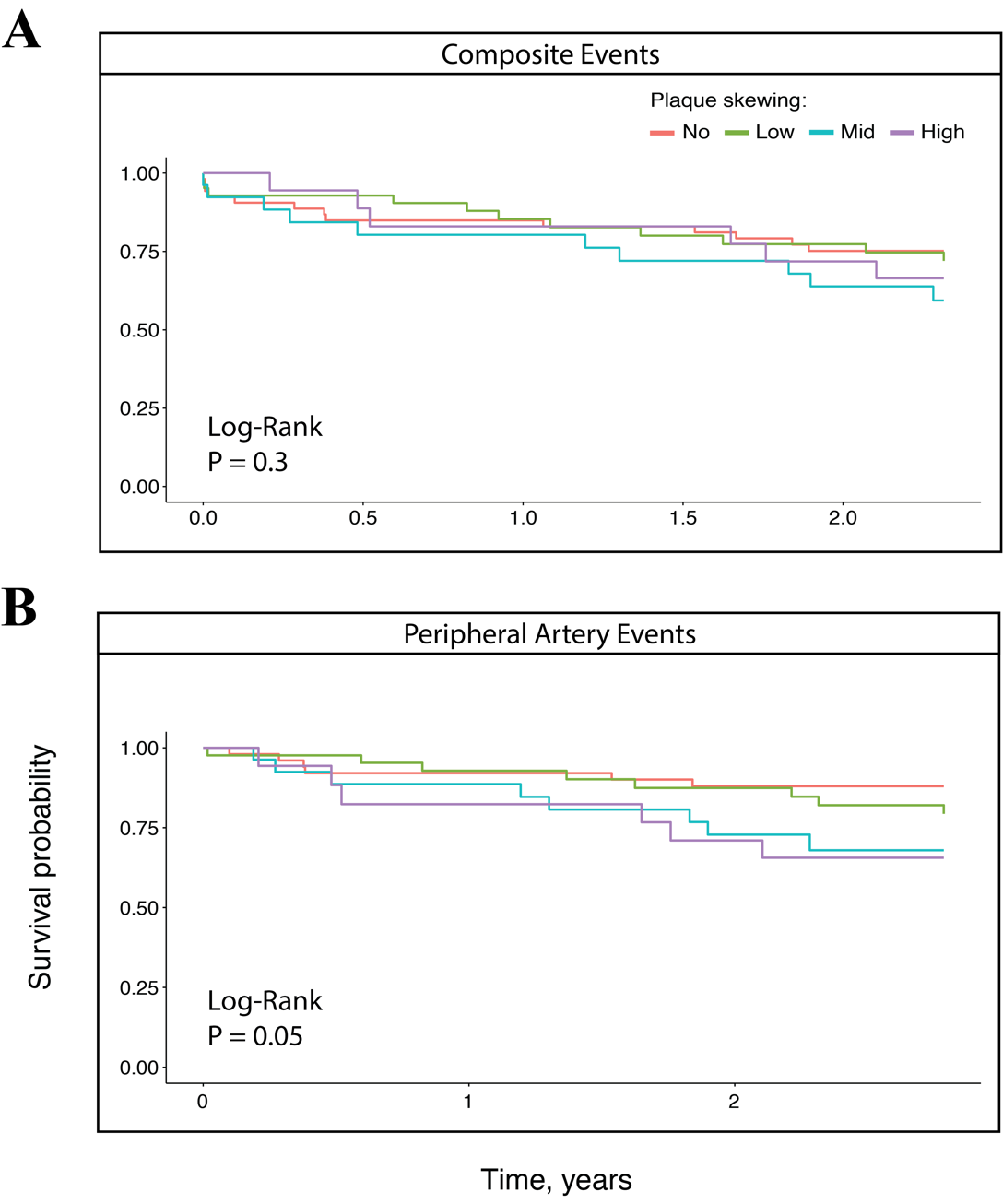


**Figure S6.** Binned plaque skewing levels, cox proportional hazards models for: (A) composite event-free survival (P = 0.3); (B) peripheral artery event-free survival (P = 0.05). Models adjusted for CAD history, BMI and current smoking. The survival probability is predicted until the occurrence of the last event.
